# Machine learning analysis plans for randomised controlled trials: detecting treatment effect heterogeneity with strict control of type I error

**DOI:** 10.1186/s13063-020-4076-y

**Published:** 2020-02-10

**Authors:** James A. Watson, Chris C. Holmes

**Affiliations:** 1 Mahidol Oxford Tropical Medicine Research Unit, Faculty of Tropical Medicine, Mahidol University, Rajvithi Road, Bangkok, 10400 Thailand; 2 Nuffield Department of Medicine, University of Oxford, Oxford, OX3 7LF UK; 3 Department of Statistics, University of Oxford, 29 Saint Giles’, Oxford, OX1 3LB UK

**Keywords:** Heterogeneous treatment effects, Randomised trials, Machine learning, Subgroup statistical analysis plan

## Abstract

**Background:**

Retrospective exploratory analyses of randomised controlled trials (RCTs) seeking to identify treatment effect heterogeneity (TEH) are prone to bias and false positives. Yet the desire to learn all we can from exhaustive data measurements on trial participants motivates the inclusion of such analyses within RCTs. Moreover, widespread advances in machine learning (ML) methods hold potential to utilise such data to identify subjects exhibiting heterogeneous treatment response.

**Methods:**

We present a novel analysis strategy for detecting TEH in randomised data using ML methods, whilst ensuring proper control of the false positive discovery rate. Our approach uses random data partitioning with statistical or ML-based prediction on held-out data. This method can test for both crossover TEH (switch in optimal treatment) and non-crossover TEH (systematic variation in benefit across patients). The former is done via a two-sample hypothesis test measuring overall predictive performance. The latter is done via ‘stacking’ the ML predictors alongside a classical statistical model to formally test the added benefit of the ML algorithm. An adaptation of recent statistical theory allows for the construction of a valid aggregate *p* value. This testing strategy is independent of the choice of ML method.

**Results:**

We demonstrate our approach with a re-analysis of the SEAQUAMAT trial, which compared quinine to artesunate for the treatment of severe malaria in Asian adults. We find no evidence for any subgroup who would benefit from a change in treatment from the current standard of care, artesunate, but strong evidence for significant TEH within the artesunate treatment group. In particular, we find that artesunate provides a differential benefit to patients with high numbers of circulating ring stage parasites.

**Conclusions:**

ML analysis plans using computational notebooks (documents linked to a programming language that capture the model parameter settings, data processing choices, and evaluation criteria) along with version control can improve the robustness and transparency of RCT exploratory analyses. A data-partitioning algorithm allows researchers to apply the latest ML techniques safe in the knowledge that any declared associations are statistically significant at a user-defined level.

## Introduction

In the medical sciences, randomised controlled trials (RCTs) provide the gold standard for evidence evaluation of novel treatments and health interventions. The growing accessibility and recording of data modalities, arising from genetics, medical imaging, mobile devices, genomics, and electronic health records captured on trial participants, alongside breakthroughs in machine learning (ML) provide new opportunities for scientific discovery of patient strata exhibiting systematic variation in treatment effect. This can improve patient outcomes and optimise treatment recommendations. However, exploratory analyses of RCTs and correct interpretations of these analyses are difficult [1, 2] and controversial [3]. Data analytic tools such as ML algorithms [4] are particularly attractive for identifying treatment effect modifiers in RCTs due to their hypothesis-free nature and ability to learn by example. Although there have been numerous recent papers on technical developments and novel methods for subgroup analysis and treatment effect heterogeneity (TEH) [5–14], we know of none to date that have considered ML paradigms purely from a testing perspective that provides strict control of the false positive rate (type I error) for the quantities we consider here, namely the evidence of crossover TEH and the evidence of predictive improvement of an ML model over a conventional statistical model. Some recent papers, e.g. [15], have derived test statistics for detecting global heterogeneity using ML, yet they lack the simplicity of our approach and the broad applied nature of our work. Moreover, we focus on detecting actionable (crossover) interactions as well as quantifying the evidence for the added predictive benefit of ML over simpler statistical models. A key component of this work is to provide concrete recommendations for how subgroup statistical analysis plans (subgroup-SAPs) can incorporate ML methods (summarised in Panel 1).

Medical statisticians know how to assess the evidence when the subgroups or interactions are predefined and the models are explicit, by counting the ‘degrees of freedom’, or number of free parameters, in the model and using formal tests of hypotheses [16–18]. But for ML algorithms the models are designed to adapt their complexity and dependency structures to the underlying problem during the training phase, and hence notions of counting parameters become meaningless. The question then remains: How to assess the true evidence of an effect following ML discovery?

We show that it is possible to train such methods, alongside conventional statistical models, to analyse RCT data and provide a global hypothesis test for the presence of TEH. The methodology explicitly uses the underlying treatment randomisation to test for TEH. We show that it is possible to formally test for the presence of patient subgroups (crossover TEH) and also formally test the added predictive benefit of the ML algorithm by ‘stacking’ the ML predictions alongside predictions from a baseline ‘vanilla’ statistical model. ML algorithms should only be used if their predictive benefit can be proven superior to that of simpler and more interpretable methods. This framework has important implications for how existing data can be used in a principled manner for trusted hypothesis generation. We hope that it will motivate careful a priori construction and monitoring of statistical analysis plans utilising the latest ML techniques. This is necessary to ensure optimal evidence evaluation and learning through retrospective discovery of TEH.

Our formal approach is illustrated step by step via an open source R Markdown computational notebook [19] which uses random forests (RF) [20] to retrospectively analyse a randomised treatment trial in severe malaria [21]; see the Methods section for further details on RF. Throughout this paper we refer to subgroup analysis and TEH interchangeably. Clinically relevant subgroups are a consequence of TEH. We take the convention that a subgroup is said to occur when the optimal treatment allocation changes, whereas heterogeneity more broadly suggests a systematic differential benefit of any one treatment. It is important to distinguish between such crossover and non-crossover TEH (see Methods), the former directly resulting in a treatment allocation that is dependent on patient characteristics [22]. Non-crossover TEH can result in patient-dependent optimal treatment allocation, but only when additional factors (e.g. cost or side effects) are brought into account to calculate the overall utility of each treatment.

## Methods

We reiterate the principle that subgroups of clinical importance identified through a retrospective data analysis, from a trial not explicitly designed to identify these subgroups, ultimately need to be validated in a focussed, independent, follow-up RCT [1]. Subgroup analysis typically exploits data from trials that were designed to answer a different primary question not involving subgroups, and hence the analysis cannot by itself provide a complete picture of the evidence. In this respect, any ML subgroup analyses should seek to establish the strength of evidence that heterogeneous treatment effects are real (true positives). Establishing and controlling the false positive rate of the discovery procedure mitigates the risk of following false leads in subsequent confirmatory trials targeting the putative subgroup, and aids in the communication of evidence from the analysis. The following sections outline a formal methodology for exploratory analysis with strict control of the type I error.

### Predefining an ML subgroup statistical analysis plan (ML subgroup-SAP)

Modern statistical and ML methods are able to automate the discovery of subgroups in high-dimensional data, and statistical scripting and programming packages such as R or Python allow the analyst to construct routines that take trial data as input and apply statistical or ML models to the data to identify potential heterogeneity. Here we consider both crossover TEH, whereby the subgroup is characterised by the set of patients predicted to benefit from a change in treatment compared to the current standard of care, and non-crossover TEH, whereby the standard of care is everywhere optimal but the benefits vary systematically across patient strata. The standard of care should be defined prospectively (before looking at the data), even if the analysis is retrospective.

In order to maintain the transparency of the evidence, an ML subgroup-SAP should be prespecified before any exploration of the primary RCT data has taken place. Failure to do so runs the risk of biasing the results [23]. When formulating the analysis plan, covering either the ML or statistical method (model) used for discovery, and the set of potential stratifying measurements used by the method, researchers should be cautioned against throwing in every possible variable and every flexible method. There is a principle here of ‘no free lunch’, or rather ‘no free power’. The choice of discovery method and the potential variables to include is an important step. Methods that trawl through measurements looking for interactions are not panaceas or substitutes for careful thought, and the more judicious the a priori data selection and choice of discovery model, the higher the expected power and ability of the analysis to detect true effects [24].

The analysis plan should also include the specification of a test statistic that can compare overall patient benefit between any two groups and that can be used to quantify the type I error when declaring beneficial subgroups. The form of this test statistic is study-specific and should relate to the clinical outcome of interest, such as survival time, cure rate, or a quantitative measurement of treatment benefit. This will typically match that used in the original study protocol of the primary trial.

### False positive control of crossover interactions: subgroup detection

**Fig. 1:**
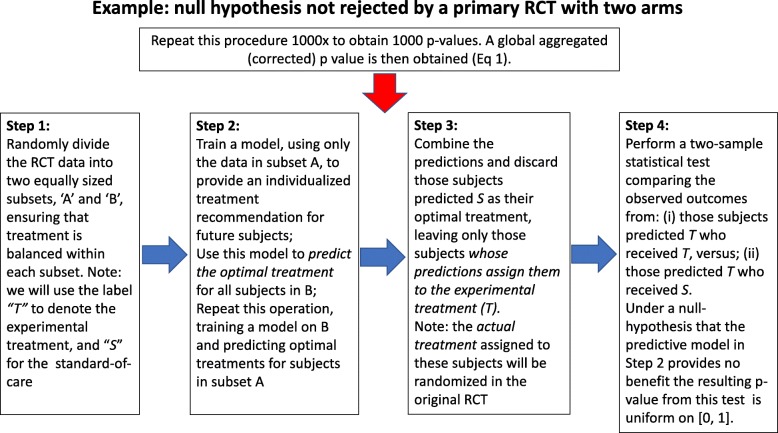
Illustrative example of hypothesis testing in exploratory subgroup discovery using 1000 iterations of twofold cross-prediction. The example considers a primary RCT with two arms where a null hypothesis of ‘no improvement from the experimental treatment’ is not rejected; i.e. there is no significant evidence of the experimental treatment providing improvement over the standard of care. Each random division results in a corresponding *p* value against the null hypothesis of no benefitting subgroup. The *p* values are then aggregated for the overall test (Eq. 1)

Subgroup detection refers to the discovery of crossover TEH whereby the optimal treatment allocation changes. We propose using a held-out data approach to construct a test for a global null hypothesis of ‘no true crossover TEH (no subgroups)’. Figure 1 illustrates this procedure using the example of a primary two-arm RCT where the original trial failed to detect an overall benefit of the experimental treatment. The approach is as follows. The trial data are repeatedly randomly divided into two subsets, with the ML method fitted independently and separately to each subset. Each ML algorithm (or statistical model), trained on one half of the data, is used to predict the individual treatment effects and thus the optimal treatments for subjects in the corresponding other half of the ‘held-out’ data, and vice versa. Combining the resulting subjects whose held-out predicted optimal treatment assignment differs from the standard of care forms a held-out subgroup of size *n*_*s*_ from the original trial of sample size *n*. The actual treatment administered to these subjects in the primary RCT is random, such that in a balanced two-arm trial we would expect half of the subjects, $\frac {1}{2} n_{s}$, to have received the standard of care and the other half the experimental treatment. This then facilitates a two-sample hypothesis test, using the test statistic defined in the analysis plan, with a null hypothesis of ‘no improved subject benefit identified through the subgroup analysis plan’. The hypothesis test compares the outcomes of the patients who were predicted to benefit from the experimental treatment and who received the experimental treatment, to those predicted to benefit from the experimental treatment but who received the standard of care. A one-sided test would be appropriate if the test statistic measures patient benefit. If there is no true benefit arising from the alternative treatment in the subgroup identified by the ML model, then the distribution of outcomes should be the same in both groups, and thus the resulting *p* value is uniformly distributed over [0,1]. If *K* iterations of this procedure are run, randomising the 50-50 data-split at each iteration, then we obtain corresponding *K* distinct *p* values {*p*_1_,..,*p*_*K*_}. We note that each of these is conservative in that the discovery model on each subset has half the sample size to identify the subgroups. Finally it is possible to form a conservative aggregated *p* value, summarising {*p*_1_,..,*p*_*K*_}, to compute a global significance test for the presence of a benefitting subgroup. This aggregation can be done by adapting a method developed for *p*-value aggregation in high-dimensional regression [25]. In brief, if *α* is the level of control of the type I error (this is usually set to 0.05), then the set of *p* values can be merged into one test using the following formula (adapted from [25]):

1 $$\begin{array}{@{}rcl@{}} p_{\text{aggregate}} = \min_{\gamma \in [\alpha,1]} \left[1, (1-\log\alpha) Q_{\gamma}\left(\{ p_{i}\}_{i=1}^{K}\right) \right], \end{array} $$

where $Q_{\gamma }\left (\{ p_{i}\}_{i=1}^{K}\right) = \min \left [ 1, Quantile_{\gamma }\left (\left \{\frac {p_{i}}{\gamma }\right \}_{i=1}^{K} \right)\right ] $. *Quantile*_*γ*_(·) computes the *γ* quantile of the set of *p* values which have been scaled by $\frac {1}{\gamma }$. This procedure sweeps over *γ*∈[*α*,1] to find the minimum value in *Q*_*γ*_. The term 1− log*α* corrects for any inflation from searching over multiple values of *γ*. Alternately the analyst could fix *γ* in the analysis plan, such as *γ*=0.5 to select the median *p* value, and then compute:

2 $$ \begin{aligned} p_{\text{aggregate}}^{(\text{median})} &= Q_{0.5}\left(\left\{\frac{p_i}{0.5}\right\}_{i=1}^{K}\right)\\ &=\min\left(1, \text{Median}[ 2p_{1},2p_{2},.., 2p_{K}]\right). \end{aligned}  $$

A proof of correctness for this aggregation procedure, for any value of *γ*∈(0,1), is provided in the supplementary Appendix, adapted from [25].

Note that if a true subgroup exists in the population from which the RCT trial participants are drawn, then $\frac {n_{s}}{n} \times 100\%$ estimates the subgroup prevalence in that population. The more refined the subgroup, the smaller *n*_*s*_ will tend to be, and hence the resulting test will have lower power to detect a true effect. That is, rarer subgroups are harder to detect. Intuitively this highlights how the original trial has reduced power to support more intricate subgroup discovery.

Optimality of this procedure is obtained when the random partitioning splits the data into two equal-size subsets. The standard error across the predictions will be proportional to $1/(\sqrt {n_{1}} + \sqrt {n_{2}})$, where *n*_1_+*n*_2_=*n* is the total trial sample size. This is minimised for *n*_1_=*n*_2_=*n*/2. We illustrate this optimality using RF applied to simulated data; see supplementary material (Additional file 1). The number of random partitions, *K*, should be chosen large enough such that the aggregate *p* value stabilises, rendering the analysis reproducible under different initial random seeds. Stability with respect to *K* can be visualised by the traceplot of the aggregated *p* value for values *k*<*K*_max_. The exact number of random splits required will depend on the context. In our simulation studies, *K*=1000 is more than sufficient, with results stabilising around *K*=200. However, an appropriate choice of *K* is context-dependent.

### False positive control of the added predictive benefit of the ML analysis

The primary outcome in a standard RCT will often be strongly associated with particular baseline covariates and prognostic factors which are predictive of the event rate, e.g. severity of disease or co-morbidities. Adjusting for these differences in baseline risk greatly enhances the power to detect subgroups of interest [26, 27]. Generalised linear models (GLMs) provide one of the most interpretable statistical model types for relating clinical outcome to a multivariate combination of prognostic factors and the randomised treatment. Using more complex and therefore less interpretable ML methods needs to be justified with respect to the added benefit over such a baseline model. In this context, the utility of ML methods is in their ability to detect non-linear interactions between prognostic factors and the randomised intervention. Using exactly the same data-splitting approach as for the discovery of statistically significant crossover subgroups, we can objectively evaluate the *added benefit* of the ML method. We illustrate the approach using a binary clinical outcome, *y*_*i*_∈{0,1} for the *i*th subject, and a logistic regression GLM, where

$$Pr(Y_{i} = 1) = \frac{\exp(Z_{i})}{1 + \exp(Z_{i})} $$

with the linear predictor *Z*_*i*_=*X*_*i*_*β*+*T*_*i*_*α*, for prognostic variables *X* and randomised treatment indicator *T*. The procedure is summarised as follows.

- For *K* iterations:
  1. Split the data into two equally sized subsets with a balanced number of treated and untreated individuals in each subset.
  2. Fit a GLM to each subset separately and record for each individual their out-of-sample linear predictor $\widehat {Z}_{i}^{GLM} = X_{i} \widehat {\beta } + T_{i} \widehat {\alpha }$, where $\left (\widehat {\beta }, \widehat {\alpha }\right)$ are obtained from the in-sample data fit.
  3. Fit the ML method to each subset separately and predict the out-of-sample outcome probabilities, $ Pr(Y_{i} = 1) = \widehat {P}_{i} = \widehat {f}_{\text {ML}}(X_{i}, T_{i}), $ to obtain the corresponding log-odds out-of-sample prediction $\widehat {Z}_{i}^{ML} = \log \left (\frac {\widehat {P}_{i}}{1 - \widehat {P}_{i}} \right) $ for each individual *i*.
  4. Fit a ‘stacked’ GLM model to the full dataset using the *n*×2 matrix of prediction values $\left (\widehat {Z}^{GLM}, \widehat {Z}^{ML}\right)$ as two independent covariate variables,
    $$Pr(Y_{i} = 1) = \frac{\exp\left(\widehat{Z}_{i}^{GLM} \theta_{GLM} + \widehat{Z}_{i}^{ML} \theta_{ML}\right)}{1 + \exp\left(\widehat{Z}_{i}^{GLM} \theta_{GLM} + \widehat{Z}_{i}^{ML} \theta_{ML}\right)} $$
    to obtain $\left (\widehat {\theta }_{GLM}, \widehat {\theta }_{ML}\right)$. Record the *p* value, *p*_*k*_, assigned to an analysis of variance (ANOVA) test for the model with *θ*_*ML*_≠0 versus a model with *θ*_*ML*_=0.
- Construct the aggregate *p* value from the set *p*_1_,..,*p*_*K*_ using the adjustment method from Eq. 1.

This method is analogous to ‘stacking’, a popular ML technique whereby multiple competing models are aggregated to form a more powerful ensemble model [28]. We propose ‘stacking’ the standard accepted ‘vanilla’ statistical model (a GLM) alongside the predictions from an ML model. The aggregate *p* value formally tests the added benefit of the ML-based predictions.

### Exploratory analysis

**Fig. 2:**
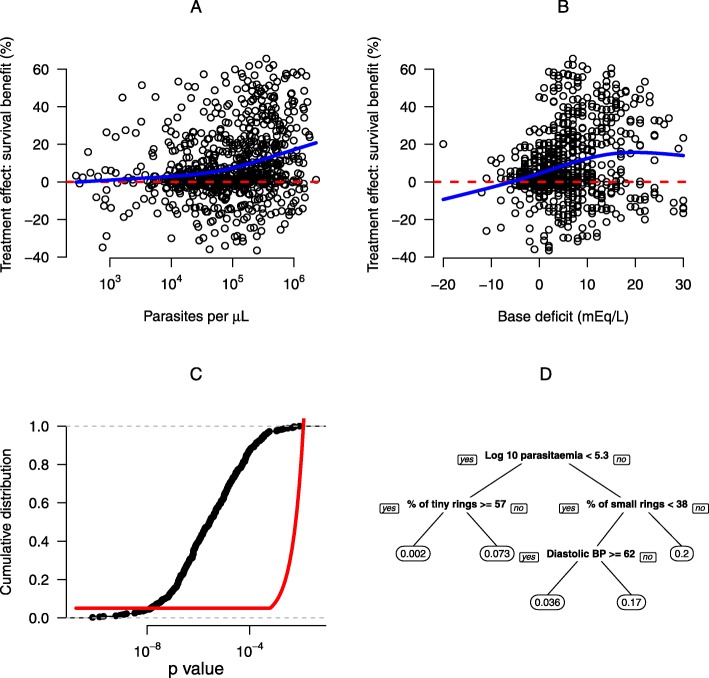
Graphical visualisation and validation of treatment heterogeneity defined by non-crossover interactions in the SEAQUAMAT trial. Panels **a** and **b** show the univariate relationships to the individual predicted treatment effect for total parasite biomass and base deficit, respectively. The *thick blue lines* show spline fits to the data. Panel **c** shows the cumulative distribution of the *p* values for the added benefit of the ML model obtained by repeated data-splitting and stacking of the standard model alongside the ML model. Significance (at the 5% level) is obtained if the *black line* crosses above the *red boundary*. Panel **d** summarises the overall non-crossover interaction found by the random forest model with a pruned regression tree model fitted to the individual treatment effects. The leaves of the tree in panel **d** show the mean treatment effect (difference in mortality between artesunate and quinine)

These ML-driven procedures for both testing the presence of crossover subgroups and for testing the added benefit of ML-predicted TEH provide valid *p* values. Under the null hypothesis, the probability of falling below the significance level *α* is upper bounded by *α*. However, this approach is by definition non-constructive: the output does not report an estimate of the discovered subgroup or an estimate of the treatment effect heterogeneity. A useful analogy is a conventional ANOVA *F* test of significance for levels of a factor. The ANOVA *F* test is an example of an ‘omnibus test’, which reports the significance (*p* value) that the outcome varies across the factors, rather than an estimate of the individual factor effects themselves. In a similar manner, our procedure simply reports a *p* value, subsequent to which further exploratory data analysis may be warranted. If the aggregated *p* value falls below a prespecified significance level, the ML model can be fit to the full dataset to estimate the individual treatment effects. This could use methods developed specifically for the determination of the structure of the TEH, e.g. [12, 14, 29], which use RF, answering questions such as: Which individuals are contained within the subgroup? Which covariates are predictive of the treatment effect heterogeneity? Is the subgroup clinically relevant? For example, this could be done via scatter plots of important covariates against the individual treatment effects. It is often possible to characterise a method detecting a true signal in the data by a few simple rules, for example using a decision tree (e.g. Fig. 2, panel D). By proceeding in this order, first evaluating the *p* value for the null hypothesis, then undertaking the exploratory analysis using the full data, formal control of the type I error is obtained.

### Transparency and reproducibility

It is essential that all the findings and analysis paths taken are transparent and auditable to an external researcher. This can be achieved through the use of statistical notebooks, akin to the laboratory notebook in experimental science. Mainstream programming environments for data analysis (such as R and Python) provide open source notebooks such as R Markdown or Jupyter which seamlessly combine the analysis and the reporting. This allows all the exploratory analysis paths to be curated. Research recorded in a computational notebook is transparent, reproducible, and auditable. Auditability can be further improved without becoming burdensome through the use of version control repositories such as *github* (https://github.com) which record, timestamp, and preserve all versions and modifications of the analysis notebooks. In this way all of the steps, time lines, and historical evolution of the subgroup analysis are included, and the work flow is open to external criticism and interrogation. Any published results can be audited back to the original RCT. Any *p* values or statistical estimates that point toward subgroup effects that are reported subsequent to the heterogeneity tests need to be clearly labelled as such and treated with caution, due to the potential for evidence inflation and post selective inference that arises from using the data twice. We prefer to label such measures that follow after data interrogation as qualitative, or *q* values, as the formal statistical sampling uncertainty is often unknown [30].

### Statistical and ML algorithms for subgroup detection

The optimal choice of statistical or ML algorithm will depend on the context of the data and on the primary endpoint of interest. When the number of candidate predictors is large but where the effects are likely to be linear, then penalised regression models such as the least absolute shrinkage and selection operator (lasso) or ridge are generally recommended [31]. An alternative, particularly if non-linear effects are expected, is random forests (RF). RF are one of the most popular and general ML methods in use today, in part as they consistently exhibit good empirical performance with little to no tuning of parameters. RF work by repeatedly building deep decision trees^1^ on bootstrapped subsamples of the data, and then aggregating predictions made by the individual trees. RF can be applied to both classification and regression. Chapter 15 of reference [31] provides a detailed overview.

In brief, the standard RF algorithm for binary classification problems proceeds as follows (for example, as implemented in the R package *randomForest*). A user-determined number of binary decision trees are constructed, where each tree is constructed independently of one another. Usually 500 trees are sufficient to obtain approximate convergence, and this is the default setting in the R package. Each tree is built on a random bootstrapped version of the training data (using sampling with replacement). At each node in the decision tree, a user-determined number of predictive variables are sampled without replacement (for classification problems the default setting is the square root of the number of available predictors). The node is then defined as the optimal data partition over all splits amongst the sampled variables with respect to a user-defined objective function (as default the Gini impurity is used for classification). The decision tree is grown until the number of training cases in each leaf reaches a lower bound (the default is 1 for classification). Note that as the training data are split at each internal node in the tree, the sample size on each branch decreases monotonically down the tree. Prediction on a new test case is done by aggregating the individual tree predictions, thus giving a classification probability. RF are also applicable to data with continuous endpoints, with extensions to survival data [32], and further extensions to the general detection of treatment effect heterogeneity [14]. Some of the well-known advantages of RF are that they are generally insensitive to the tuning parameters used in the model (e.g. the number of trees, the parameters governing the depth of trees), and they can implicitly handle missing values. In our illustrative application, we use RF with the default parameter settings from the R package *randomForest*. This analysis can be exactly replicated using the compute capsule available on *Code Ocean* [19], and readers are encouraged to play with the default parameter settings should they wish to explore further.

## Results

### ML-driven exploratory RCT subgroup analysis

Panel 1 summarises how ML methods can be used for exploratory analyses testing for the presence of significant crossover TEH which results in statistically significant subgroups. The framework we propose is novel, and it relies on recent results in the statistics literature for aggregating correlated *p* values into a single, reproducible *p* value for the null hypothesis ‘no crossover TEH’. The core of the framework relies on random data-splitting and cross-prediction, leading to unbiased optimal treatment predictions. To increase transparency, we recommend using computational notebooks to document the process, ideally prespecified via an ML subgroup-SAP. In the following we illustrate how this framework is applied to a large randomised treatment trial in severe malaria, the analysis of which provides an open source computational template for ML exploratory subgroup analysis [19].

### Antimalarial pharmacodynamics of artemisinin in severe malaria

Severe *Plasmodium falciparum* malaria is a medical emergency with case fatality rates ranging from 10 to 40% [33]. A recent major advance in the treatment of severe malaria has been the introduction of parenteral artesunate. In Asia, this has been shown to reduce mortality by a third [21], and in Africa by a fifth [34]. To illustrate the methodology advocated in this work, we use data from the definitive study of artesunate for severe malaria in Asia (SEAQUAMAT: South East Asian Quinine Artesunate Malaria Trial). This was a large multi-country randomised trial comparing intravenous quinine to intravenous artesunate [21].

The superiority of parenteral artesunate for severe malaria is now well established [35]. Thus, in this retrospective analysis the artesunate arm is considered ‘standard of care’. The complete statistical analysis is published as an open source *Code Ocean* capsule and is entirely reproducible [19]. This analysis provides an easily adjusted template for new exploratory subgroup analyses of different datasets.

We chose to use RF to fit the data, one of the most popular and important ML methods in use today [20]. The RF method deals well with multiple correlated covariates, as is the case in these data. We first evaluate whether there is evidence for a subgroup of patients who would benefit from quinine treatment as opposed to artesunate. The subgroup analysis does not reject the null hypothesis of ‘homogeneous optimal treatment allocation’ (*p*=1), showing that there is no evidence in the data of any subgroup benefitting from quinine.

This analysis was followed by examining the added benefit of the predictive RF ML model relating patient survival to the baseline measurements and treatment. An aggregation of the *p* values obtained by repeated data-splitting and ‘stacking’ of the out-of-sample ML model predictions alongside the validated best linear predictor (the linear predictor on the logistic scale comprising Glasgow coma scale, base deficit, and treatment [36]) showed a strongly significant added benefit of the RF ML model (*p* = 10^−6^, Fig 2, panel C). The statistical significance of the repeated data-splitting and cross-prediction procedure can be assessed visually by comparing the cumulative distribution of the resulting *p* values against the boundary curve as given by Eq. 1.

Further exploratory analysis attempted to characterise possible interactions explaining this variation in predicted individual treatment effect. This analysis showed that significant TEH can be partially explained by the total non-sequestered parasite biomass (panel A) and the base deficit (panel B). This treatment heterogeneity can be summarised using a pruned classification and regression tree (CART) model decision tree (panel D). This suggests that the greatest benefit of parenteral artesunate (estimated as 20 percentage points difference in mortality) is seen in patients with large numbers of circulating young ring stage parasites (an interaction between total parasitaemia and % of young rings). This is not highlighting a clinically relevant subgroup, but it helps elucidate the mechanism of action of artemisinin, a useful exercise in light of emerging drug resistance [37]. Moreover, these results are concordant with the current proposed mechanism of action of the artemisinin derivatives and the importance of the artemisinin-specific mode of action in the treatment of severe malaria. Artemisinin derivatives kill a broader range of parasite stages compared to quinine, notably the younger circulating ring forms, thereby reducing further sequestration and subsequent death in patients with a high parasite biomass [38].


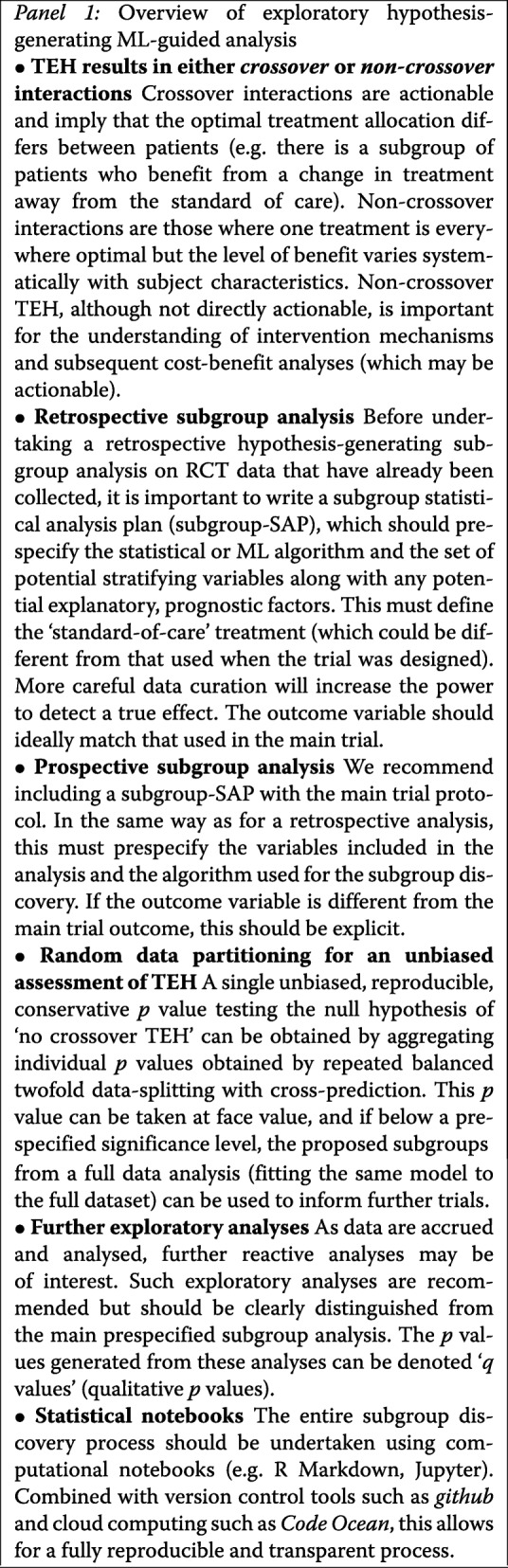


## Discussion

This work demonstrates how modern machine learning algorithms can be trained safely to discover treatment effect heterogeneity in a way that rigorously controls for type I error. The validity of our data-splitting and cross-prediction procedure holds irrespective of the method used, provided that samples are independently recruited from the study population—the same assumption necessary for the validity of cross-validation methods. If this is not the case, for example if patients are recruited in pairs, or are related in some manner, then adjustments need to be made to ensure that the *p* value reports the correct out-of-sample evidence. The choice of discovery algorithm should depend on the measurement variables collected (how many, and of which type) and the primary or secondary outcomes of the study for which subgroup analysis is to be applied, e.g. survival time, binary outcome, continuous risk score. The specification of the stratifying measurements used by the method needs careful thought under a principle of ‘no free power’ in that feeding in irrelevant predictor variables will reduce the ability to detect true signals [24].

The approach we advocate here is generic. Exploring the benefit of one predictive model over another, either traditional or machine learning, can be done within a common statistical machine learning analysis plan, where the null hypothesis is that Model B provides no additional benefit in prediction over that of Model A. In our corresponding compute capsule available on *Code Ocean* [19], we implemented a test for the added benefit of random forests over a generalised linear model, and the reader can easily adapt this code to compare other models, traditional or otherwise, as long as each model can provide a prediction of the outcome following treatment.

It is important that the analysis is transparent, that the methods, data transformations, and analytic procedures are laid out and documented in an auditable plan, and that any code base used is properly documented and available for scrutiny. We recommend the use of open source repositories such as *github* or cloud computing services such as *Code Ocean* for fully reproducible data analyses. By following some simple guidelines, we hope to improve upon the reliability and stability of subgroup analysis reported in the literature. Recent advances in statistical machine learning algorithms along with recent advances in measurement technologies have the potential to impact heavily and positively in the advancement of medical science. However, alongside these advances great care must be taken to ensure that the integrity of the statistical analysis and the validity of the evidence base are upheld at all times.

## Supplementary information

**Additional file 1** Proof of correctness for *p* value adjustment.

## Data Availability

All data are available via *Code Ocean* at the following url: https://codeocean.com/capsule/2760408/tree/v2.

## Abbreviations

GLM: Generalised linear model
RCT: Randomised controlled trial
RF: Random forest(s)
ML: Machine learning
ML subgroup-SAP: Machine learning subgroup statistical analysis plan
TEH: Treatment effect heterogeneity

## Footnotes

1 Figure 2, panel D gives an example of a shallow decision tree. In contrast, RF build deep decision trees from subsamples of the data where the branches (questions) descend until only a small number of samples lie within each leaf of each tree. Predictions on new data are then averaged across all trees.
