# Supplementary material for: Machine learning analysis plans for randomised controlled trials: detecting treatment effect heterogeneity with strict control of type I error
Source: Trials. 2020 Feb 10;21:156. doi: 10.1186/s13063-020-4076-y (PMC7011561; doi:10.1186/s13063-020-4076-y)
Supplement: Supplementary file 1 — Additional file 1 Proof of correctness for p value adjustment. [file 13063_2020_4076_MOESM1_ESM.pdf]

Machine learning analysis plans for randomised controlled trials: identifying treatment effect heterogeneity with strict control of type I error

James A Watson[1,2,*] and Chris C Holmes[2,3,*]

[1]Mahidol Oxford Tropical Medicine Research Unit, Mahidol University, Bangkok, Thailand
[2]Nuffield Department of Medicine, University of Oxford, Oxford, UK
[3]Department of Statistics, University of Oxford, Oxford, UK

*Correspondence: jwatowatson@gmail.com; cholmes@stats.ox.ac.uk

A Proof of correctness for p-value adjustment

This adapts the proof given in[1]. Meinshausen et al give proofs of correctness for their procedure but are more complicated given that the setting is high-dimensional regression and the proofs concern the family-wise error rate and the false discovery rate when the number of predictor variables is large. In our setting there is a single null hypothesis: no treatment effect heterogeneity (in their setting there is a hypothesis for each variable in the regression model) and therefore it is possible to greatly simplify the notation. However, we note that the essence of the proof is almost identical.

Recall that for K random splits of the data we obtain K p-values: $p_1, .., p_K$. Recall that the γ-adjusted global p-value is given by $Q(\gamma) = q_\gamma\left(\{p_k/\gamma\}_{k=1}^K\right)$, where $q_\gamma(\cdot)$ is the empirical γ-quantile function (this drops the min function for simplicity).

Define the random variable $\pi(u)$ as the fraction of the p-values p_k that are less than or equal to u:

$$\pi(u) = \frac{1}{K}\sum_{k=1}^{K} \mathbb{1}_{\{p_k \leq u\}}$$

Note that the events $\{Q(\gamma) \leq \alpha\}$ (the event: the γ-quantile of the $1/\gamma$ inflated p-values is less than α) and $\{\pi(\gamma\alpha) \geq \gamma\}$ (the event: a proportion larger than γ of the p-values p_k are less than $\gamma\alpha$) are equivalent:

$$P\left[Q(\gamma) \leq \alpha\right] = P\left[\pi(\gamma\alpha) \geq \gamma\right]$$

Using the Markov inequality,

$$P\left[\pi(\gamma\alpha) \geq \gamma\right] \leq \frac{1}{\gamma} E[\pi(\gamma\alpha)]$$

By the definition of $\pi(\cdot)$,

$$\frac{1}{\gamma} E[\pi(\gamma\alpha)] = \frac{1}{\gamma}\frac{1}{K}\sum_{k=1}^{K} E\left[\mathbb{1}_{\{p_k \leq \gamma\alpha\}}\right]$$

Under the null hypothesis (no treatment effect heterogeneity), the p-values p_k are uniformly distributed over the interval $[0,1]$, hence:

$$\frac{1}{\gamma}\frac{1}{K}\sum_{k=1}^{K} E\left[\mathbb{1}_{\{p_k \leq \gamma\alpha\}}\right] = \frac{1}{K\gamma} K\gamma\alpha = \alpha,$$

which completes the proof.

References

[1] Nicolai Meinshausen, Lukas Meier, and Peter Bühlmann. P-values for high-dimensional regression. *Journal of the American Statistical Association*, 104(488):1671–1681, 2009.
